
JOURNAL INFORMATION
==============================
NLM Title Abbreviation: Halls J Health
NLM Journal ID: 0011644

Hall's Journal of Health

Publisher: Biomedical Journal Digitization Project

ARTICLE INFORMATION
==============================
PMCID: PMC9184075
PMID: 36486011
Article version: 1
Subjects: Articles

Editorial Notice

Print publication date: 1873 Oct
Volume: 20
Issue: 10
First page: 245
Last page: 245
License: This work is free of known copyright restrictions. For more information, please see PMC Back Issue Digitization.
License URL: https://creativecommons.org/publicdomain/mark/1.0/

==============================

### EDITORIAL NOTICE.

Graham Flour.—No more pernicious custom exists than that of sacrificing the most nutritious portion of the wheat in the process of converting it into flour. The grain of wheat, chemically, is a miniature man. Before using it for food we bolt out the brains, bones and nerves—and if the missing elements were not supplied from other articles of diet, the most deplorable consequences would ensue. When those king structures, the bones, brains and nerves are poorly nourished, other organs, notably the lungs, show the general blight first; and it is a significant fact that consumption is always preceded and accompanied by a species of anemia, which the rejected portion of wheat, could it be properly digested and assimilated, is precisely adapted to cure. If bread made, as Nature intended it should be, from all the constituents of wheat, was as universally used as that from its white emasculated residue, called "superfine flour," a few generations would see consumption and scrofula, with their legion of kindred ills, less prevalent. In looking for a reliable article of "Graham flour," containing all the nutritious elements of the best wheat, we have been much pleased with that manufactured by James Campbell, of Rochester, New York, called "Carr's Graham Flour."

---

**WATERS' CONCERTO PARLOR ORGANS**

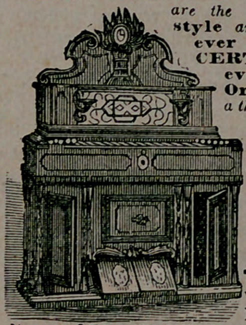

*are the* **most beautiful** *in* **style** *and* **perfect** *in* **tone ever made.** *The* **CONCERTO STOP** *is the* **best ever placed in any Organ.** *It is produced by a third set of reeds* **peculiarly voiced,** *the* **EFFECT** *of which is* **MOST CHARMING** *and* **SOUL STIRRING,** *while its* **IMITATION** *of the* **HUMAN VOICE** *is* **SUPERB.** *Terms liberal.*

**A GREAT OFFER.** HORACE WATERS & SON, 481 Broadway, N. Y., *will dispose of* **100 PIANOS** *and* **ORGANS** *of* **first-class makers,** *including* **WATERS',** *at* **extremely low prices for cash,** *or* **part** *cash, and balance in* **small monthly** *payments.* **New 7-Octave first-class PIANOS, all modern improvements,** *for* **$275** *cash. Organs* **$55. $75. DOUBLE-REED ORGANS, $100; 4-STOP, $110; 8-STOP, $125,** *upwards. ILLUSTRATED CATALOGUES MAILED for one stamp. A large discount to Ministers, Churches, Sunday-Schools, Temperance Societies, Lodges, etc.* **AGENTS WANTED.**
